# Novel adaptive immune systems in pristine Antarctic soils

**DOI:** 10.1038/s41598-024-83942-y

**Published:** 2025-01-18

**Authors:** Marc W. Van Goethem, Oliver K. I. Bezuidt, Rian Pierneef, Surendra Vikram, David W. Hopkins, Thomas Aspray, Grant Hall, Stephan Woodborne, Ian D. Hogg, Trent R. Northen, Weidong Kong, Daniele Daffonchio, Don A. Cowan, Yves Van de Peer, Manuel Delgado-Baquerizo, Thulani P. Makhalanyane

**Affiliations:** 1 Department of Biochemistry, Genetics and Microbiology, University of Pretoria, Pretoria, 0028 South Africa; 2 Biological and Environmental Sciences and Engineering Division (BESE), King Abdullah University of Science and Technology, 23955-6900 Thuwal, Saudi Arabia; 3 Department of Biochemistry, Genetics and Microbiology, Faculty of Natural and Agricultural Sciences, University of Pretoria, Hatfield, Pretoria, 0028 South Africa; 4 Department of Biochemistry, Genetics and Microbiology, Faculty of Natural and Agricultural Sciences, DSI/NRF SARChI in Marine Microbiomics, University of Pretoria, Hatfield, Pretoria, 0028 South Africa; 5 Scotland’s Rural College, West Mains Road, Edinburgh, EH9 3JG UK; 6 School of Energy, Geoscience, Infrastructure and Society, Heriot-Watt University, Edinburgh, EH14 4AS UK; 7 Mammal Research Institute, University of Pretoria, Private Bag X20, Hatfield, 0028 South Africa; 8 iThemba LABS, Private Bag 11, Johannesburg, 2050 South Africa; 9 Canadian High Arctic Research Station, Polar Knowledge Canada; and School of Science, University of Waikato, Waitkato, New Zealand; 10 Molecular EcoSystems Biology Division, Lawrence Berkeley National Laboratory, 1 Cyclotron Rd, Berkeley, CA 94720 USA; 11 State Key Laboratory of Tibetan Plateau Earth System and Resources Environment, Institute of Tibetan Plateau Research, Chinese Academy of Sciences, Beijing, 100101 China; 12 Department of Plant Biotechnology and Bioinformatics, Ghent University, 9052 Ghent, Belgium; 13 Center for Plant Systems Biology, VIB, 9052 Ghent, Belgium; 14 Bioinformatics Institute Ghent, Ghent University, 9052 Ghent, Belgium; 15 Laboratorio de Biodiversidad y Funcionamiento Ecosistémico, Instituto de Recursos Naturales y Agrobiología de Sevilla (IRNAS), CSIC, Seville, Spain; 16 Unidad Asociada CSIC-UPO (BioFun), Universidad Pablo de Olavide, Seville, Spain; 17 Department of Microbiology, Faculty of Science, Stellenbosch University, Stellenbosch, 7600 South Africa; 18 The School for Data Science and Computational Thinking, Stellenbosch University, Stellenbosch, 7600 South Africa

**Keywords:** Adaptive immunity, Antarctica, Antiphage,, Bacteria, CRISPR-Cas, Evolutionary drivers, Metagenomics, Microbiome, Bacteriophages, Soil microbiology

## Abstract

Antarctic environments are dominated by microorganisms, which are vulnerable to viral infection. Although several studies have investigated the phylogenetic repertoire of bacteria and viruses in these poly-extreme environments with freezing temperatures, high ultra violet irradiation levels, low moisture availability and hyper-oligotrophy, the evolutionary mechanisms governing microbial immunity remain poorly understood. Using genome-resolved metagenomics, we test the hypothesis that Antarctic poly-extreme high-latitude microbiomes harbour diverse adaptive immune systems. Our analysis reveals the prevalence of prophages in bacterial genomes (Bacteroidota and Verrucomicrobiota), suggesting the significance of lysogenic infection strategies in Antarctic soils. Furthermore, we demonstrate the presence of diverse CRISPR-Cas arrays, including Class 1 arrays (Types I-B, I-C, and I-E), alongside systems exhibiting novel gene architecture among their effector cas genes. Notably, a Class 2 system featuring type V variants lacks CRISPR arrays, encodes Cas1 and Cas2 adaptation module genes. Phylogenetic analysis of Cas12 effector proteins hints at divergent evolutionary histories compared to classified type V effectors and indicates that TnpB is likely the ancestor of Cas12 nucleases. Our findings suggest substantial novelty in Antarctic cas sequences, likely driven by strong selective pressures. These results underscore the role of viral infection as a key evolutionary driver shaping polar microbiomes.

## Introduction

Understanding the ecological role played by viruses in altering ecosystem processes through their influence on both the phylogenetic and functional diversity of their hosts remains a major ecological endeavour^1–5^. In soils, viruses affect the diversity and abundance of microorganisms^6–9^ thereby influencing processes such as nutrient cycling and carbon sequestration^10–13^. Their role as mediators of ecosystem services is pronounced in the poly-extreme environments of the McMurdo Dry Valleys of Eastern Antarctica where prokaryotes govern nutrient cycling. While Antarctic soils harbour diverse microbes and viruses^14–17^, host-virus interactions remain relatively unexplored. This is despite the fact that viral infections may pose direct threats to microorganisms by influencing nutrient/biomass turnover, and on ecosystem functioning^18^. However, few studies have assessed these relationships, especially in poly-extreme environments, such as Antarctica, where microbial communities disproportionately influence ecosystem functions. Likewise, understanding the range of defence mechanisms used by microbes, to avoid viral infections, is crucial^19–22^.

A notable prokaryotic defence mechanism, the CRISPR-Cas (clustered regularly interspaced short palindromic repeats—CRISPR associated) system^23–27^, allows microorganisms to specifically target and degrade viral DNA or RNA^28–33^. CRISPR-Cas systems are widespread accessory elements across bacterial and archaeal plasmids^34,35^, and have been the subject of extensive studies in the last decade owing to their efficacy in genome editing^33,36,37^. Consequently, the repertoire of known CRISPR-Cas systems has expanded significantly in terms of both quantity and diversity^35,38–41^. These systems recognize the foreign DNA of an invading phage and cleave sequences from its genome, which become integrated as spacers within the host’s CRISPR array^42^. The CRISPR-Cas system thus provides signatures of previous infection events by retaining the ‘genomic scars’ of historical infections^43^. We hypothesise that this prokaryotic immune system may be more pronounced in poly-extreme ecosystems, where evolution is remarkably constrained and under strong selective pressures due to several persistent abiotic stressors. Because CRISPR-Cas systems represent an ancient adaptive immune strategy in prokaryotes^44^ elucidating this antiviral defence pathway may reveal the immunological memories within bacterial genomes from pristine Antarctic soils. However, the extent to which these mechanisms may influence the diversity and function of terrestrial Antarctic soil communities remain poorly understood.

The Mackay Glacier region in Antarctica is one of the most remote and challenging poly-extreme environments on Earth where multiple extreme conditions including sub-zero temperatures, very low nutrient status and an absence of precipitation, render soils virtually inhospitable^45–47^. Previous, studies have revealed remarkable insights regarding the phylogenetic diversity of microbial communities in Mackay Glacier soils. For instance, these soils are predominately composed of members of the *Acidobacteriota* and *Bacteroidota* phyla^48^. These groups are known to encode a suite of antibiotic resistance genes, which may hint at possible adaptive strategies for survival in the low pH and oligotrophic conditions typical of these soils^16^. Other studies have shown that several taxa affiliated with a novel family of group 1 l [NiFe]-hydrogenases which seemingly contribute to water generation through trace gas scavenging^45^. We have also documented a diverse array of tailed bacteriophages (i.e. dsDNA phages) in these soils^14^, showing that their distribution is substantially influenced by both soil pH and site altitude.

While the diversity and evolution of defence mechanisms in this ecosystem have not been studied previously, a recent studies on rock-associated Antarctic communities in the Miers Dry Valley have shown that poly-extreme environments harbour diverse defence systems^15^. The results of these studies suggest the presence of several innate immune systems including BREX (BacteRiophage EXclusion) and DISARM (Defence Island System Associated with Restriction-Modification), which were the predominant modes of antiphage immunity employed by bacteria in Antarctic desert hypoliths^15^. However, hypoliths represent only a small fraction of the Dry Valley terrain, and there is a distinct lack of studies focused on exposed surface soils.

Our overarching hypothesis is that the pristine soil microbiota of the Mackay Glacier region harbour novel adaptive immune systems. These novel systems are detectable through CRISPR-Cas arrays, which have evolved in response to the selective pressures from viral infection. Here, we aim to provide insights on adaptive immune systems through (i) characterizing the CRISPR-Cas systems within bacterial genomes, and (ii) investigating their role in viral defence mechanisms. By studying the presence of CRISPR-Cas systems in this unique ecosystem, we hope to gain a better understanding of the mechanisms by which microorganisms protect themselves from viral infections in extreme environments. We further predict that the CRISPR-Cas systems in these Antarctic soils may play crucial roles in protecting microorganisms from viral infections and maintaining the stability of the ecosystem. Using a genome-resolved metagenomic analysis, we provide the first insights of bacterial adaptive immunity, and bacterial-viral associations in Antarctic soils.

## Results and discussion

### Strong evidence of predation-prey associations in Antarctic soils

**Fig. 1:**
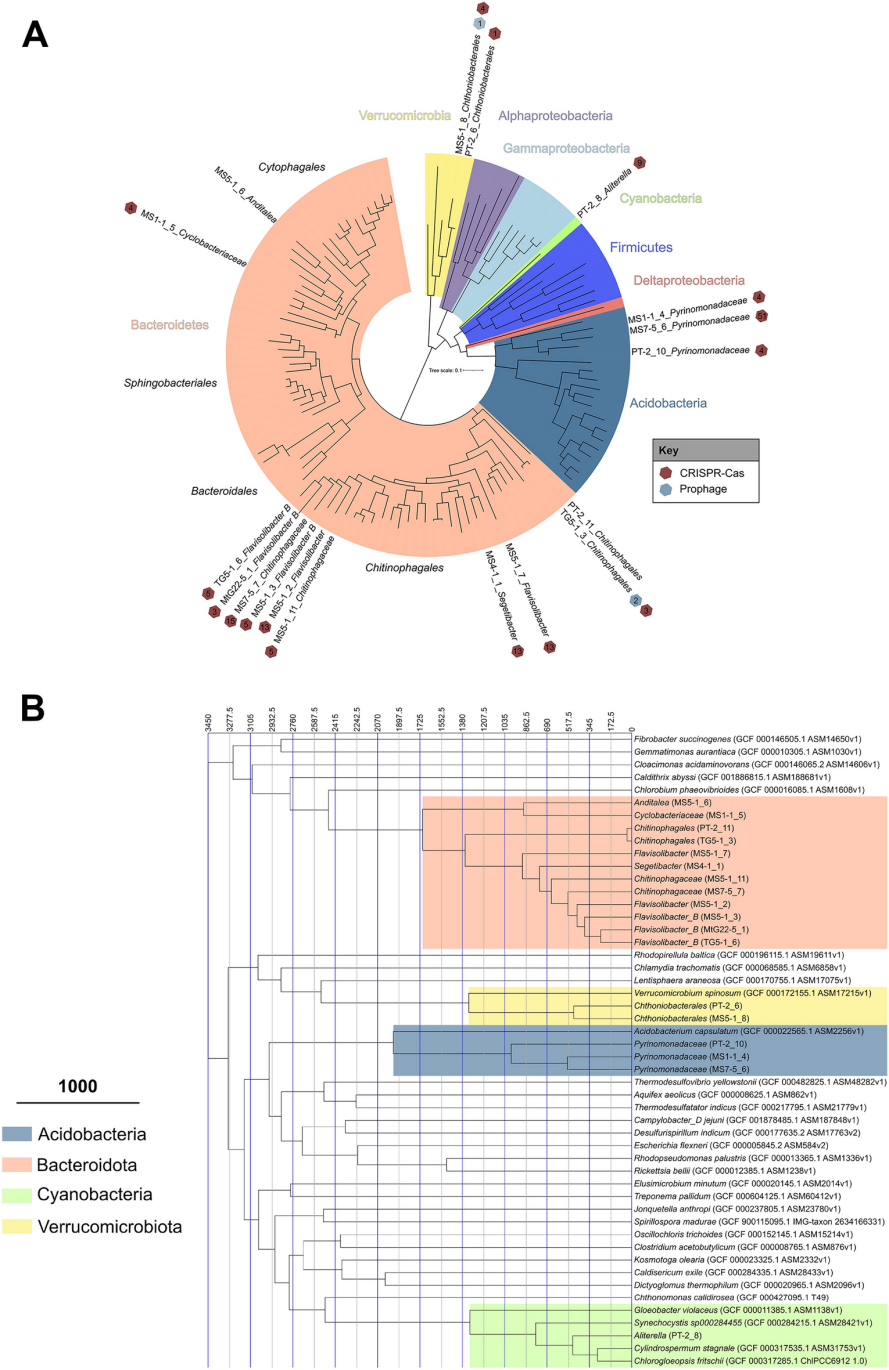
(**A**) Maximum likelihood tree of all metagenome-assembled genomes (MAGs). Phylogenetic tree constructed with a concatenated alignment of 49 core, bacterial genes. The tree includes 18 bacterial MAGs from this study that are aligned to 100 known reference genomes present in the RefSeq database. Hexagons adjacent to the genome names indicate either the presence of a CRISPR-Cas repeat (maroon) or a prophage (grey) within the host genome. Numbers in hexagons indicate counts of prophages or CRISPR spacers. (**B**) Bayesian divergence estimates of our Antarctic MAGs placed among 32 reference outgroup genomes. Time scale is estimated in mega-annum.

Our study expands current insights regarding the genetic mechanisms explaining prey-predator co-evolutionary associations between bacteria and their viruses in poly-extreme Antarctic conditions (Supplementary Table 1). Following metagenome sequencing, assembly, and genome binning (Supplementary Tables S2 and S3), we recovered 18 medium- to high-quality metagenome-assembled genomes (MAGs) (Fig. 1a). We note that this is a small sample size of reconstructed genomes, and likely reflects an underestimate of our sequencing effort (Supplementary Fig. 1). Thus, we predict that the rate of gene discovery from these sequence data would increase with higher sequencing depth. Notably, we only recovered approximately 69% of available sequence diversity from sample TG5-1, although other depth curves were approaching saturation at 12 million sequences (Table 1).

**Table 1:** Genomic statistics for the 18 MAGs recovered from the Mackay Glacier region of Antarctica.

Bin Id	Lineage (GTDB-Tk)	Genome Size (bp)	Completeness (%)	Contamination (%)	Prophages	CRISPR spacers
MS1-1.4	*Pyrinomonadaceae*	32,91,545	82.38	6.46	0	4
MS1-1.5	*Cyclobacteriaceae*	33,19,274	75.94	4.27	0	4
MS4-1.1	*Segetibacter*	26,59,359	58.37	3.45	0	13
MS5-1.11	*Chitinophagaceae*	38,56,181	94.88	2.96	0	5
MS5-1.2	*Flavisolibacter*	33,35,429	97.49	2.96	0	13
MS5-1.3	*Flavisolibacter_B*	16,54,061	73.45	1.72	0	5
MS5-1.6	*Anditalea*	30,47,815	51.02	1.72	0	0
MS5-1.7	*Flavisolibacter*	31,49,465	78.72	1.23	0	13
MS5-1.8	*Chthoniobacterales*	26,52,899	66.01	6.45	1	4
MS7-5.6	*Pyrinomonadaceae*	39,55,314	91.10	9.47	0	51
MS7-5.7	*Chitinophagaceae*	34,32,039	91.89	9.28	0	15
MtG22-5.1	*Flavisolibacter_B*	28,54,497	50.86	3.45	0	3
PT-2.10	*Pyrinomonadaceae*	30,82,920	53.69	3.59	0	4
PT-2.11	*Chitinophagales*	25,44,403	57.68	4.16	0	0
PT-2.6	*Chthoniobacterales*	24,95,165	89.53	5.17	0	1
PT-2.8	*Aliterella*	23,33,589	64.44	3.45	0	9
TG5-1.3	*Chitinophagales*	16,45,326	83.62	6.45	2	3
TG5-1.6	*Flavisolibacter_B*	26,76,035	82.79	6.16	0	5

These MAGs include three *Acidobacteriota*, one *Cyanobacteria*, twelve *Bacteroidota* and two *Verrucomicrobiota*, representing both dominant, and rare bacterial phyla in these soils (Supplementary Note 1 and Supplementary Fig. 1). The genome sizes of these bacteria ranged from 2.7 – 5.9 Mb, when accounting for completeness. These genomes had moderately low G + C contents (mean = 42.8%, range 35.14%—61.44%), which is surprising given the expectation that extreme environments may select for organisms with high G + C content that allow for more stable DNA structures due to the molecular interactions of base stacking^49,50^.

We estimated the genome replication rates for each MAG^51^ and found that the highest genome replication rates were associated with *Acidobacteriota* (mean = 3.06) and the *Verrucomicrobiota* (mean = 2.90). These differed compared with *Bacteroidota* (mean = 2.48) and *Cyanobacteria* (2.04). Estimating the minimal doubling time with codon usage bias^52^ suggested very low division times in the Antarctic bacteria (slow-growing bacteria; > 5 h), with only certain members of the *Bacteroidota* predicted to double within five hours. Specifically, the cyanobacterial genome (> 21 h) and the Verrucomicrobiota genomes (> 15 h) indicated very low division times. Overall, these patterns suggest extremely slow growth rates which is expected given the poly-extreme conditions in this region (see additional information regarding the climatic conditions and these taxa in Supplementary Note 1). Given the low division rates, we believe that it becomes reasonable to predict that the slow evolutionary processes acting on microbial communities in this environment are likely to delay selection. There is some support for this assertion including the divergent ecological patterns of Antarctic soil microbiota^53,54^ and the substantial differences in community composition compared with soils from outside the continent^45^.

Our study provides strong evidence that Antarctic bacterial communities may have ancient origins. Bayesian evolutionary analysis, used to produce time-measured phylogenies, suggests that the genomes retrieved from our studies ranged between 500 and 1,200 Mya (Fig. 1b). These findings are consistent with previous estimates of cryptoendolith divergence in Antarctica^55^. The results also support approximations for other genomes retrieved from Antarctic soils^45^. Altogether, the unique monophyletic clades of our Antarctic MAGs were distinct—suggesting that these bacteria diverged from other known taxa during the Precambrian (541 Mya). Taxonomic analysis indicated that 12 of our MAGs potentially represent novel species as they show low homologies to those available in reference databases. Considering this evidence, we predict that bacteriophages associated with these bacteria may have also been co-separated from other microorganisms for a similar length of time since host-virus specificity is mostly strain specific. We hypothesized that this distinct, and specific, co-evolution may be corroborated by the recovery of uncharacterized and potentially novel adaptive immune signatures in Antarctic host genomes (see Supplementary Note 2 on viral sequence analyses).

### CRISPR-Cas systems provide evidence of virus attacks in Antarctica

**Fig. 2:**
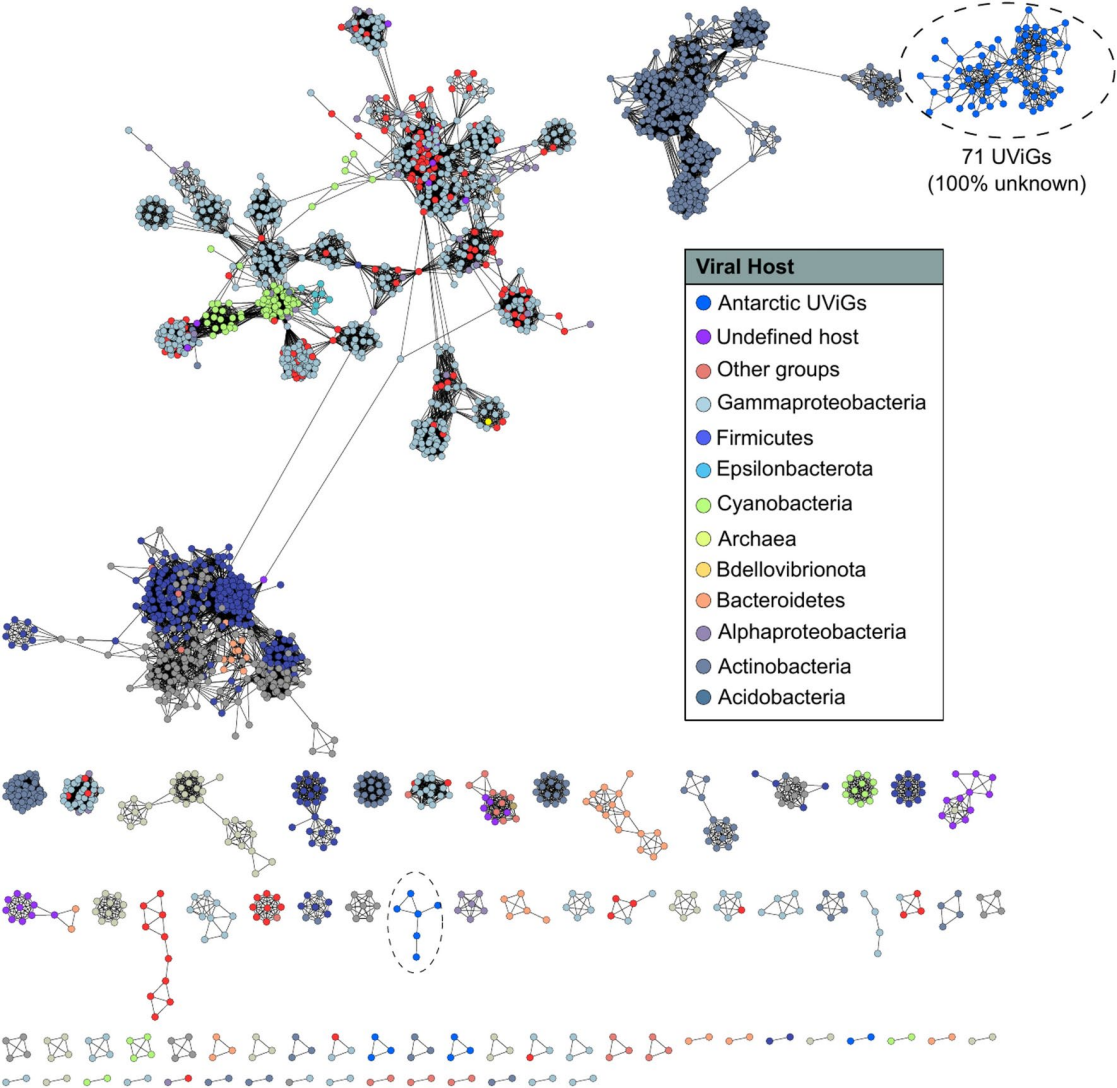
Viral contigs in MAGs and metagenomic datasets. Viral protein cluster network (PC) produced using Cytoscape v3.8.2. Network showing the clustering of recovered viral genomes and viral genome fragments with known RefSeq viral genomes (v85) based on their shared predicted protein content. Major viral families (nodes) are indicated by the colour of their host, with vOTUs found in this study indicated by blue circles and with dotted lines. The edges (lines between nodes) indicate significant shared protein content.

The detection of prophages in MAGs and AMGs on phage contigs (Supplementary Note 2) supports the notion of unique host-virus histories, as almost all our Antarctic viral genomes are completely unrelated to known viruses based on protein similarities (Fig. 2), as observed in many unexplored habitats previously^56–58^. The results from this study suggest that the host adaptive immune system, associated with divergent microbiota in Antarctica soils, may be more prominent than initially envisaged. However, apart from previous studies on rock associated microbiota, there is a severe knowledge deficit regarding host adaptive immune systems associated with poly-extreme environments.

To further explore host-virus histories associated with our MAGs, we searched for related diverse defence strategies against phage predation. As part of determining the adaptive immune system, we found putative CRISPR-Cas systems in 16 of the 18 MAGs (*ca*. 89%). In terms of CRISPR arrays, the identified conserved repeats ranged from 23 to 30 bp across four of the retrieved genomes. The repeats were flanked by unique spacers (obtained from viruses), that were an average of 36 bp in length (range 34—38 bp). The largest set of CRISPR cassettes was found in the MS7-5_6 genome (*Acidobacteriota*), with 51 CRISPR unique spacers housed between 6 different CRISPR cassettes. These values are within the optimal number of spacers, previously suggested to range between 10 – 100 spacers within bacterial genomes^59^. The CRISPR loci within bacterial genomes retain the memory of past viral infections and foreign DNA encounters^59,60^, suggesting at least 50 previous such events. Yet, the length of these loci appears to be directly related to the capacity to respond to an infection^61^. In other words, there appears to be a trade-off between maintaining a vast genetic memory of attacks (harbouring more spacers) and the functionality of the CRISPR mechanism^59^. One hypothesis for this is that array size represents a balance between maintaining immunity to many potential threats (older infections) and updating immunity to contend with new threats^62^. The remaining genomes only had between three and 16 spacers, which is more similar to human gut microbes (average of 12 spacers)^63^ than the average cassette size of between 20 and 40 spacers^64^. We speculate that the lower spacer count may be due to limited encounters with a small set of phages. In this scenario, the spatial constraints of the soil microhabitat limit the number of potential interactions between phages and putative hosts. This suggests that phage diversity may be low in this region of Antarctica. Not only are cells immobilized by adsorption to soil particles of the Antarctic desert pavement, but rarely, if ever, subject to precipitation events which may allow for the mobilization of cells or virus-like particles, thus reducing the spectrum of infection events considerably.

**Fig. 3:**
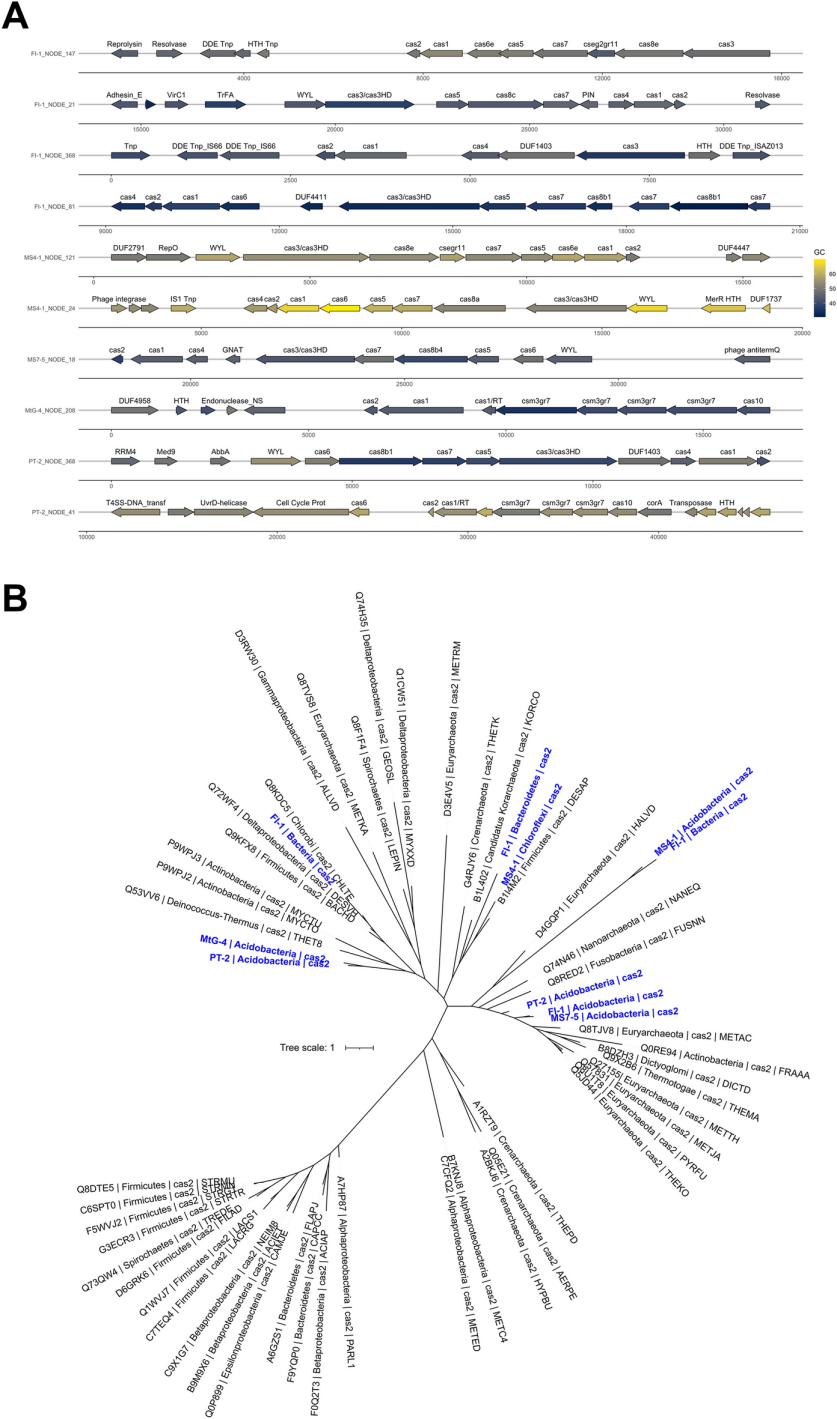
Cas proteins revealed varied taxonomic histories. (**A**) The contigs containing Cas genes are shown. They are colored by their G + C content which showed substantial variation across the contigs. (**B**) Phylogenetic tree of Cas2 genes recovered from our metagenomes (shown in blue) with reference sequences in black. Blue labels indicate both the sampling site and taxonomy. Tree scale is shown on the left.

In addition to the CRISPR-Cas cassettes, 16 MAGs had relatively low abundances of *cas* genes, with between 6 and 42 loci per MAG. These *cas* genes constituted 122 unclassified sequences (*n* = 221 total *cas* sequences), followed by several classified sequences including 48 type III, 31 type I, 20 type IV and 2 type V Cas systems. These Cas types are similar to those previously reported in Antarctic surface snow in which CRISPR-cas types I, II and III were most common^65^. The MS7-5_6 MAG (*Acidobacteriota*) had a contig with 10 genes associated with a hybrid CRISPR-Cas Class I system. This contig also had a GCN5-related N-acetyltransferase (GNAT) toxin domain^66^ (see Fig. 3a), which functions by acetylating charged tRNA molecules to prevent translation. Previous studies suggest that these GCN5-related N-acetyltransferase toxin domains may represent novel substrates for several enzymes linked to antibiotic modification^67^.

We further investigated unbinned metagenomic contigs, which possessed eight or more co-localized *cas* genes, to determine if they represented novel CRISPR-Cas variants. Taxonomically, the CRISPR-Cas systems recovered from these contigs were affiliated members of the *Acidobacteriota* (*n* = 6 contigs), Unclassified Bacteria (*n* = 2), *Chloroflexota* (*n* = 1) and *Bacteroidota* (*n* = 1). However, the taxonomic relationships of these taxa suggest potentially shared histories with a variety of bacterial phyla (Fig. 3b). The architecture of effector complexes, within the CRISPR-Cas systems, suggests that most of these were class 1 with type I or type III systems. Genes for Cas1 and Cas2 proteins were ubiquitously distributed across all contigs (Fig. 3a). These genes were always structured as Cas1-Cas2 complexes. In four examples, the Cas1-Cas2 complex was flanked upstream by *cas*4, which directly interacts with the Cas1-Cas2 complex, to process pre-spacers prior to integration as the Cas4-Cas1-Cas2 complex^68^. However, in two instances, our analyses revealed that *cas*4 was downstream of the Cas1-Cas2 complex, which is an unconventional arrangement of these genes based on data from previous studies^33^. In all 10 cases, the effector genes were located upstream of the Cas1-Cas2 operon.

The remaining four CRISPR-Cas systems may represent novel variants, based on arrangements of their effector modules (Fig. 3b)^33,69^. These results imply ongoing horizontal gene transfer and recombination events of diverse CRISPR-Cas loci, probably led by continuous interactions with the same viruses. Notably, these uncategorized CRISPR-Cas system types were affiliated with members of the *Acidobacteriota*. They include FI-1_NODE_368 (cas2-cas1-cas4-cas3), which lacks an effector complex and seems to be closely related to Type IU. Contig FI-1_NODE_81 (cas4-cas2-cas1-cas6-cas3-cas5-cas7-cas8b1-cas7-cas8b1-cas7) a potential Type I-B variant based on multiple copies of cas7 and cas8 at the terminus of the array. Contig MtG-4_NODE_208 (cas2-cas1-cas1-RT-csm3gr7-csm3gr7-csm3gr7-cas10), potentially a Type IIIU array with three copies of csm3gr7, and finally, contig PT-2_NODE_41 (cas6-cas2-cas1-csm3gr7-csm3gr7-csm3gr7-cas10) which may be a Type IIIA variant or Type IIIU variant since it lacks csm2, csm4 and csm5 genes.

All 10 predicted CRISPR-Cas systems were associated with CRISPR arrays. These systems were composed of spacers that ranged from 2 to 122 bp in length, with an average length of 35 bp. The *cas*2 sequences showed some divergence from those previously reported, and these results contrasted with our expectations. Instead, the *cas*2 sequences clustered among unrelated phyla, in some cases grouping within the domain *Archaea* (Fig. 3a). These results are not surprising given the fact that these genes are known to be horizontally acquired. This may indicate that the *cas*2 gene is not always taxonomically conserved. Instead, the result suggests mobilization via inter-phylum horizontal gene transfer (HGT) events or evidence of phylum-specific *cas* subtypes. A recent study showed that CRISPR-Cas systems may contribute to the propagation of transposable elements by facilitating transposition into specific sites^70^. Similarly, our results support previous reports since we found transposase elements on almost half of the 10 CRISPR-Cas-containing contigs analysed.

**Fig. 4:**
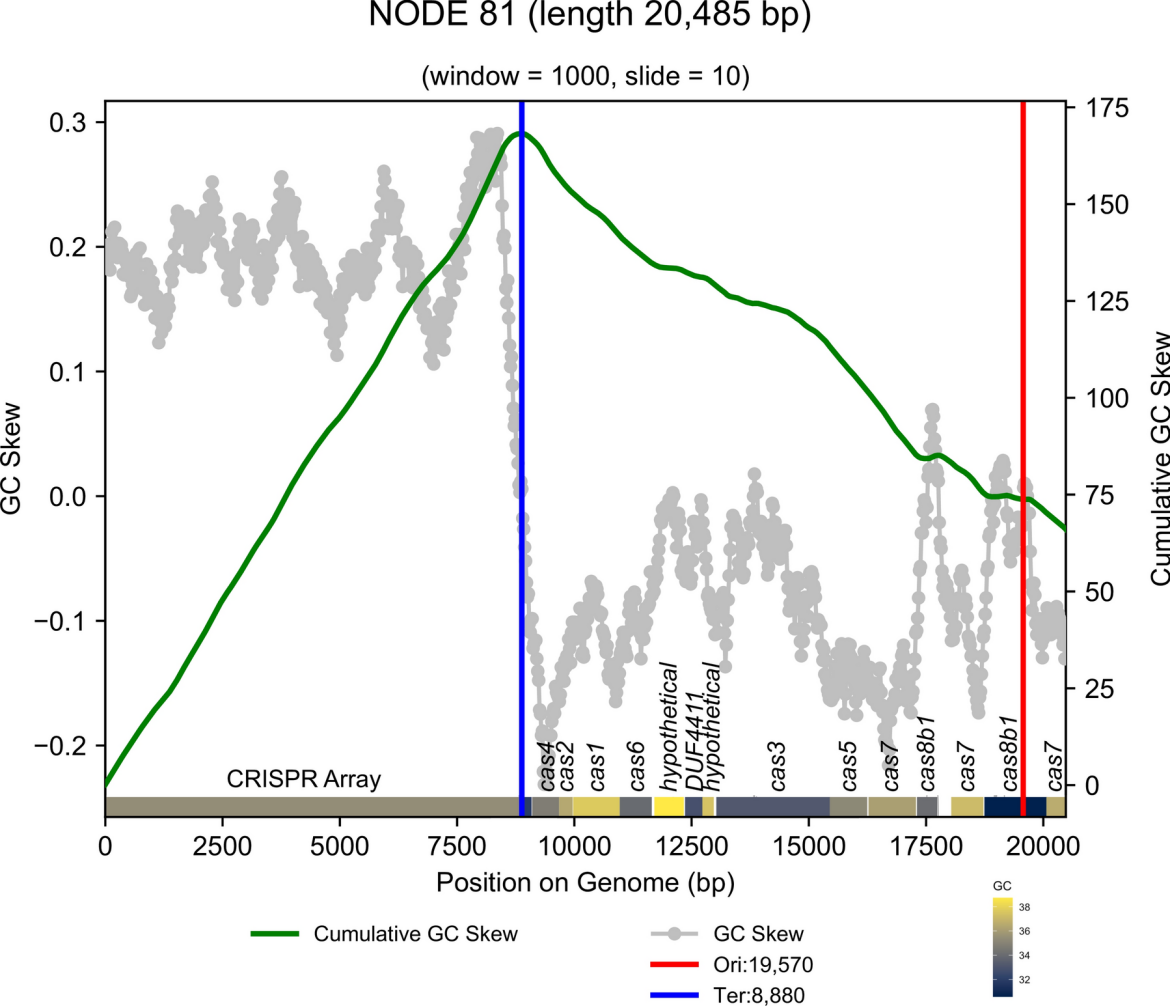
Plot indicating GC skew across contigs. G + C skew across NODE_81 is shown along the contig (x-axis plots gene position in the contig) with G + C skew on the left-hand y-axis (grey points) and Cumulative G + C skew on the right-hand y-axis (green line). The predicted origin and termination of replication are shown in red and blue lines, respectively. The CRISPR-Cas array is shown at the bottom of the figure with the G + C content of each gene shown.

Based on these data, we speculated that these Antarctic CRISPR-Cas systems were horizontally transferred as ancient mobilization events. This suggestion is supported by an evaluation of the G + C skew, among the 10 contigs containing *cas* genes, as a proxy for the timing of insertion events^71^. Here, we inferred HGT through the detection of strong deviations in G + C content for a genomic fragment compared to the remaining genomic signature. Specifically, on NODE_81 from the FI-1 metagenome, the G + C content over the Cas proteins varied minimally across each gene yet is markedly different from the G + C content of the CRISPR array upstream of the *cas* genes (Fig. 4). By contrast, the contigs containing integrated prophages within the microbial genomes showed very high variations in G + C content (i.e. G + C skew) across the contig which hints at their foreign origin^72^ (Supplementary Fig. 3). Our Bayesian diversity estimates also indicated ancient divergence events of our MAGs from known bacteria. It is thus likely that the phages of these bacteria have similarly ancient Precambrian histories, which offers a possible explanation for their unique gene compositions.

**Fig. 5:**
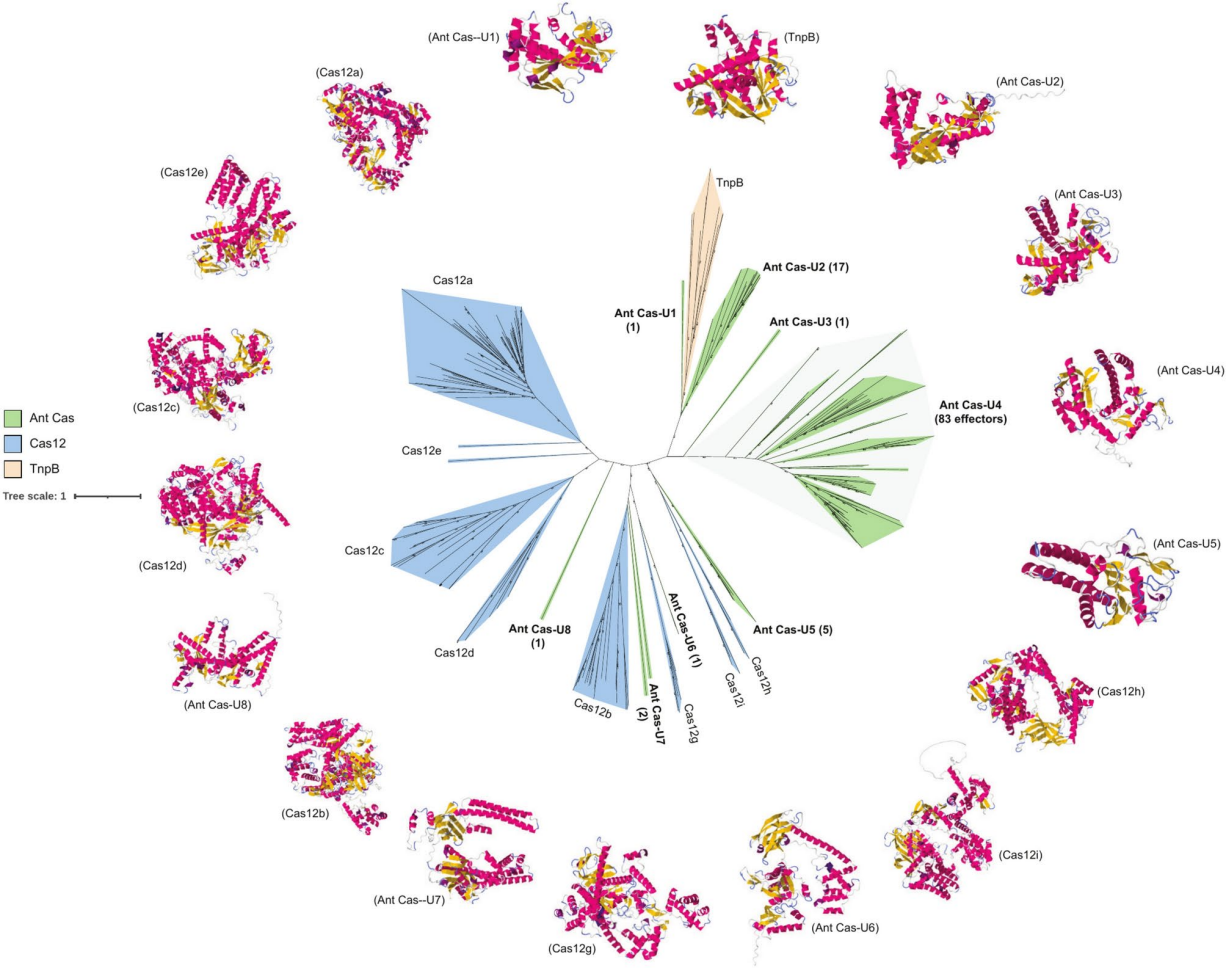
A novel subgroup of cas12-like effectors identified from pristine Antarctic soils. Novel Antarctic cas12 effectors (Ant Cas-U1—Ant Cas-U8; shown in green) show unique phylogenies from known cas12 effectors (Cas12a-Cas12i; blue). The clades Ant Cas-U1 and Ant Cas-U2 show a shared evolutionary history with TnpB nucleases (orange) and have similar predicted tertiary protein structures (outer ring).

Following this, we explored our data for the diversity of type V CRISPR-Cas systems. From the data, we identified a total of 216 contigs longer than 1 kb from 16 of the 18 metagenomes with predicted cas12 effectors proteins. Of these, 112 contigs with sizes ranging from 1,007 to 48,306 bp that possessed non-partial cas12 proteins were retained for downstream analyses. The lengths of effector proteins in these contigs varied from 89 to 630 amino acids, and this contrasted with previous reports that have indicated the average lengths for type V associated effector proteins to be ~ 400 amino acids and longer^73,74^. As effector proteins associated with type V CRISPR-Cas systems are mainly distinguished by the possession of a RuvC nuclease domain, we also found these to be characteristic of our 111 effectors, including the smallest (89 aa) putative cas12 protein. Only one of these lacked a RuvC domain but instead possessed a rudimentary helix-turn-helix domain. Further inspection of contigs possessing these indicated that only 13 of our effectors were proximal to CRISPR arrays, and unlike typical CRISPR-Cas systems none of the 112 were co-localized with the cas1-cas2 complex. Phylogenetic analysis of these indicated that just nine of our effectors (Ant Cas U5-8) clustered with previously characterized cas12 effectors. We then observed that 18 of our other effectors indicated a close phylogenetic relationship with transposon encoded TnpB proteins, suggesting that the Antarctic type V effectors may have evolved from TnpB associated nucleases, which has been speculated previously^75^. We observed a further 83 additional effectors from our data that formed a distinct clade (indicated as Ant Cas U4), potentially representing a novel subgroup of cas12-like effectors (Fig. 5).

Altogether, we speculate that the unique diversity of the genes found in these Antarctic soils may be the result of a ‘slowed down’ evolution of genes selected during warmer periods of time. The Antarctic continent was a temperate rainforest during the mid-Cretaceous period ~ 140 Mya^76^ and we speculate that the subsequent cooling of the continent may have constrained evolutionary forces from acting at their previous pace. Combined, these lines of evidence point to an ancient, acquired immunity of bacteria in Antarctica while contemporary infection events continue to occur through lysogenic phage infections.

## Conclusions

We used metagenomes from remote and pristine Antarctic soils to assess their viral and bacterial diversity. Multiple lines of evidence suggest extensive phage-host interactions, potentially novel viral diversity, and CRISPR-Cas variants. The phage signatures (vOTUs) were linked to the infection of dominant soil bacterial lineages in these surface soils, including members of the *Bacteroidota* and *Acidobacteriota*, while prophages embedded within *Verrucomicrobiota* and *Bacteroidota* MAGs offer further insight into contemporary infections. CRISPR-Cas systems, part of the bacterial adaptive immune system, were common to 4 of 18 MAGs analyzed, indicating acquired immunity in both *Bacteroidota* and *Acidobacteriota*. Additional Class I CRISPR-Cas arrays (types I-B, I-C and I-E) were detected in the assembled metagenomes, where four CRISPR-Cas arrays did not match existing architectures and thus may be novel variants. The difference in the architecture observed within our CRISPR-Cas arrays suggests that these may provide an enhanced ability for bacteria to resist viral predation and adsorption. This may be a result of extreme conditions in the Antarctic which may influence viruses to employ different infection strategies, and expand on the diversity and versatility of CRISPR-Cas systems^77^. Our analysis of G + C content and GC skew across CRISPR-Cas contigs showed low variations in G + C skew in CRISPR-Cas arrays, but more variation in prophages, suggesting that these acquired immunity markers are ancient whereas proviral elements appear to be the result of recent foreign DNA transfer. This is further evidenced by the description of novel, Antarctic-exclusive cas12-like effectors with remarkable sequence homology to TnpB transposases, suggesting that TnpB could be an ancestor of Cas12 nucleases adopted by CRISPR-Cas systems.

## Materials and Methods

### Sample collection and preparation

**Fig. 6:**
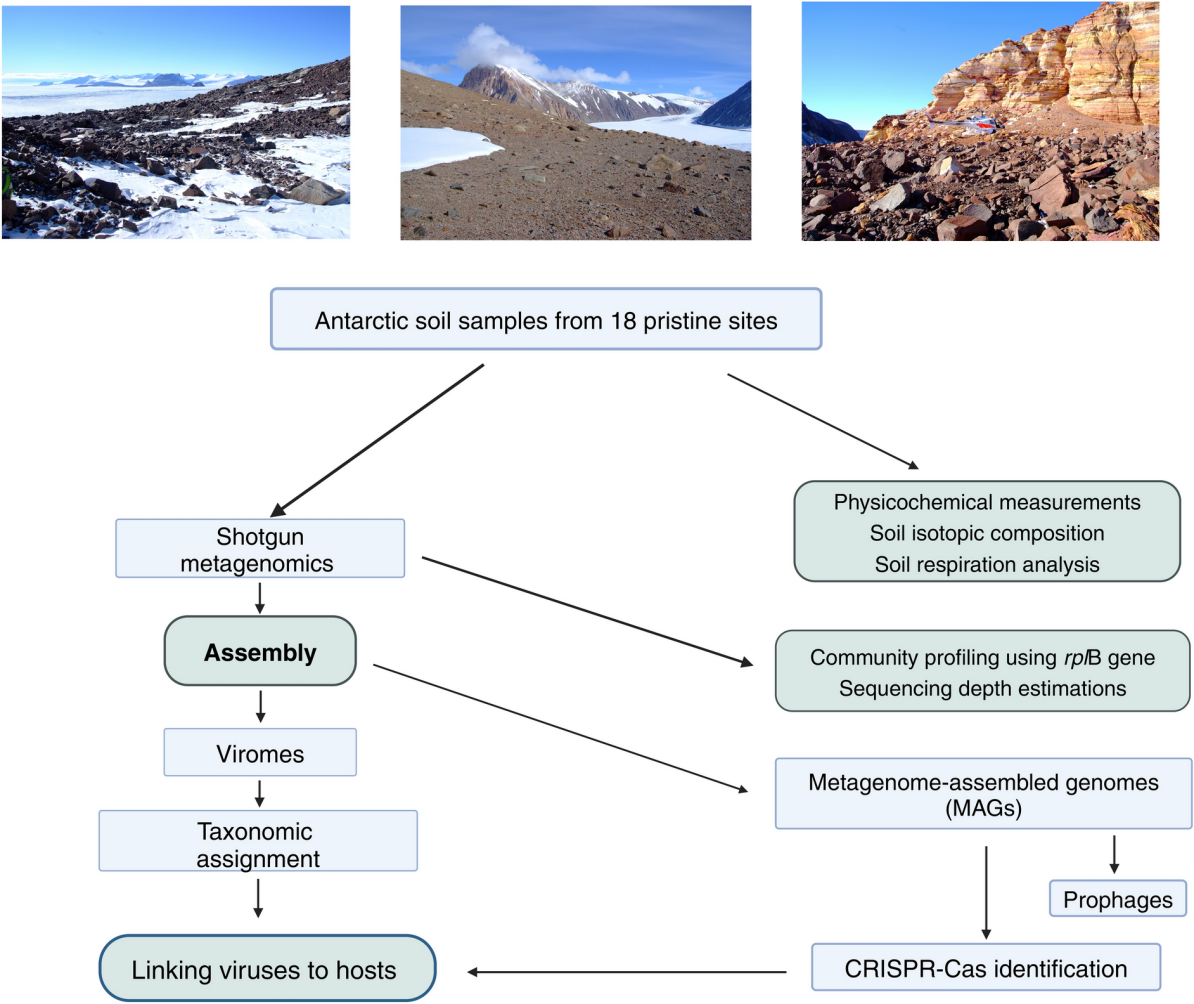
Sampling sites and methodology. Photographs showing three of the ice-free Antarctic sampling sites, from left to right; Mount Gran, Mackay Glacier site 3, and Pegtop Mountain (credit Prof. Don Cowan). Flow diagram indicates the broad methodology used in this study.

Surface soils were collected from 18 remote sites in Eastern Antarctica, between the Mackay Glacier (76.52°S 161.45°E) and the Drygalski Ice Tongue (Fig. 6)^16^. Methods of DNA isolations, soil physicochemical analyses, soil isotopic measurements and soil respiration experiments have been reported previously^48^. Briefly, the Mackay Glacier—located to the north of the McMurdo Dry Valleys, Victoria Land, Antarctica—were sampled for surface mineral soil samples from 18 ice-free sites. At each of the 18 sites, approximately 20 g soil samples were retrieved aseptically from five positions within a 1 m^2^ quadrat (0–5 cm soil depth), providing 90 soil samples in all. Soils were stored in sterile Whirl Pak bags (Nasco International, WI, USA) on ice during sampling and transport in the Antarctic, and at—80 °C in the laboratory (Centre for Microbial Ecology and Genomics, University of Pretoria, South Africa) until processed. After DNA extractions, metagenomes were sequenced on an Illumina HiSeq 2000 instrument producing 250 bp paired-end libraries. Sequencing depth was determined using Nonpareil v3.301^78^.

### Metagenome analysis and assembly

All 18 metagenomes were filtered to cull low-quality reads and those containing ambiguous bases (internal *N*’s) using Prinseq-lite v0.20.4^79^. Further filtering parameters included removing the sequencing adapters, reads with Phred scores < 20 for six consecutive bases, unpaired reads, and reads shorter than 100 bp in length. All Illumina PhiX sequences were identified and removed using BBDuk^80^ to eliminate the potential of contaminating viral signals in our analysis^81^. We determined the microbial taxonomy of each sample using SingleM, which relies on analyses of universal single-copy ribosomal subunit proteins (*rplB*), rather than the 16S rRNA gene to infer taxonomy (https://github.com/wwood/singlem). Each filtered metagenome was individually assembled using metaSPAdes v3.12^82^ under default settings with *k*-mer step increases from 21 to 141. We used MicrobeCensus v1.1.0 to calculate the number of genome equivalents in each sample^83^.

### Reconstruction of microbial genomes

From the 18 assemblies (one from each site), we reconstructed microbial genome bins using MetaBAT 2 v2.12.1^84^. All contigs > 1,500 bp in length were retained and depth coverage information was obtained using BBMap^80^ by mapping corresponding metagenomic reads back to those contigs. The bins were assessed for completeness using CheckM2 v1.0.2^85^ and metagenome-assembled genomes (MAGs) that were > 50% complete and were < 10% contaminated were retained for further analyses. Next, we queried indicators of genome quality, such as the presence of 5S, 16S and 23S ribosomal subunit genes and the presence of at least 18 unique tRNAs according to MIMAG standards^86^. Taxonomy was then assigned using the Genome Taxonomy Database Toolkit (GTDB-Tk) v2.3.2 release version 214^87^. CARD RGI was used to determine the prevalence of antibiotic resistance genes (ARGs) as hallmarks of resistance to bacterial antibiotic production^88^. The 18 retained MAGs were inspected for CRISPR-Cas repeats using MinCED^89^ and for prophages (integrated viral genomes) using VirSorter v1.0.6^90^. A maximum likelihood phylogenomic tree, based on 49 core bacterial genes from our 18 MAGs and 100 reference genomes (RefSeq database), was built using FastTree2 v2.1.10. Phylogenomic trees were visualized in iTOL v6.34^91^. We used iRep to estimate bacterial replication rates^51^ and gRodon2 to calculate growth rate^52^.

### Viral taxonomic analysis

We also explored each metagenomic assembly for bacteriophages using VirSorter v1.0.6^90^. Contigs were manually inspected for viral “hallmark” genes from categories 1 and 2 (complete viral contigs), and 4 and 5 (prophages). The quality of the predicted viral contigs was assessed using CheckV v1.0^92^. Contigs > 10 kb that were thought to be viral were then clustered using vConTACT2 v0.9.19^93^ to establish a network of protein clusters among known phages from the Virome database. The edges of the network are significant gene-sharing similarities between contigs, which are represented as nodes. These uncultivated viral genomes (UViGs) were also inspected for possible auxiliary metabolic genes (AMGs) that could have been acquired from their host. We built phylogenetic trees using MAFFT v7.294b^94^ of the AMGs identified in UViGs and their homologs in host MAGs to determine the possible origin of the gene. Finally we used the dbCAN2 web server^95^ to identify glycosyl hydrolases (GH) and glycoside transferases (GT) in the MAGs. UViGs were clustered into viral OTUs (vOTUs) at 95% average nucleotide identity (ANI) and 85% alignment fraction (AF).

### CRISPR spacer analysis and protein structure analysis using AlphaFold2

Metagenomes and MAGs were explored for the presence of adaptive immunity systems such as CRISPR-cas gene types using hmmsearch with (E-value 1e^−05^) against Cas gene profiles obtained from a study by Makarova, Wolf, Iranzo, Shmakov, Alkhnbashi, Brouns, Charpentier, Cheng, Haft and Horvath^33^. These were further assessed for the presence of innate immune response genes using RPS-BLAST (E-value 1e^−05^) against conserved domain databases (CDD) of clusters of orthologous groups (COGs) and protein families (Pfams)^96^. Results from these searches were manually filtered for the identification of CRISPR-Cas systems, toxin-antitoxins (TA), restriction-modification (RM), bacteriophage exclusion (BREX), abortive infection (Abi), defense island system associated with restriction-modification (DISARM), and other recently identified systems using a refined list of COG and Pfam identifiers reported to be associated with these defense mechanisms^33,97^.

Cas reference sequences were extracted from UniProtKB/Swiss-Prot (https://ftp.uniprot.org/pub/databases/uniprot/current_release/knowledgebase/complete/uniprot_sprot.fasta.gz; accessed 2021/05/25); a high-quality, manually annotated and non-redundant protein sequence database^98^. For each Cas protein family the reference amino acid sequences and our amino acid sequences were included in multiple sequence alignment using Mafft v7.294b with the “linsi” parameter specified^94^. Trees were constructed based on the multiple sequence alignments using IQ-TREE v2.1.2^99^ with the “MFP” parameter invoked which uses ModelFinder^100^ to determine the most appropriate model. Tree refinement was performed in iTOL v6.3^91^. For the Cas G + C plots we used the R v4.0.2 statistical environment while the G + C skew was calculated using iREP v1.10^51^. Cas protein structures were determined using AlphaFold2^101^.

### Bayesian analysis

Divergence time was estimated using Bayesian analyses. A multiple protein sequence alignment was constructed using GTDB-Tk v.1.7.0^87^ and was based on the 120 GTDB core bacterial marker genes. The alignment included a set of 32 reference outgroups previously used to calibrate the crown bacteria^45,55^. BEAUti v.1.10.4^102^ was used to specify parameters for Markov chain Monte Carlo (MCMC) tree analyses in BEAST v.1.10.4^103^. The multiple protein sequence alignment was imported and a Gamma Site Model with four categories was selected. The LG amino acid substitution model was used and a Relaxed Clock model with a Log Normal distribution. A Coalescent Constant Population tree prior was chosen and bacterial divergence (crown) was calibrated using a prior on the root of 3,453 million years ago (Ma) and a standard deviation of 60 Ma^45,55,104^ with a Log Normal distribution specified. MCMC parameters were set at 10,000,000–40,000,000 Chain Lengths with a sampling frequency of 1,000. Tracer v.1.7.2^105^ was used to assess convergence with burn in percentages between 10 and 80 to obtain the optimal effective sample size. A Maximum clade credibility tree and Mean heights were selected to produce a summarized target MCMC tree with TreeAnnotator v.1.10.4^103^. iTOL was used for final tree visualization and analysis^91^.

## Supplementary Information

Supplementary Information 1.

Supplementary Information 2.

## Data Availability

The quality-filtered, unassembled metagenomic sequences are available on the MG-RAST server under the accession numbers 4667018.3 to 4667036.3. All contigs longer than 200 bp from the assembled metagenomes are deposited on the NCBI under the BioProject PRJNA376086. Code for statistical analyses is available at https://github.com/SAmicrobiomes/.
