# Supplementary material for: Novel adaptive immune systems in pristine Antarctic soils
Source: Sci Rep. 2025 Jan 18;15:2368. doi: 10.1038/s41598-024-83942-y (PMC11742911; doi:10.1038/s41598-024-83942-y)
Supplement: Supplementary file 1 — Supplementary Information 1. [file 41598_2024_83942_MOESM1_ESM.docx]

**Supplementary Material**

**Novel adaptive immune systems in pristine Antarctic soils**

Marc W. Van Goethem^1,2^, Oliver K. I. Bezuidt^3,4^, Rian Pierneef^3,4^, Surendra Vikram^1^, David W. Hopkins^5^, Thomas Aspray^6^, Grant Hall^7^, Stephan Woodborne^7,8^, Ian D. Hogg^9^, Trent R. Northen^10^, Weidong Kong^11^, Daniele Daffonchio^2^, Don A. Cowan^1^, Yves Van de Peer^1,12,13,14^, Manuel Delgado-Baquerizo^15,16^, and Thulani P. Makhalanyane^3,4*^

**Affiliations:**

^1^ Department of Biochemistry, Genetics and Microbiology, Centre for Microbial Ecology and Genomics, University of Pretoria, Pretoria, 0028, South Africa

^2^ King Abdullah University of Science and Technology, Biological and Environmental Sciences and Engineering Division (BESE), Thuwal 23955-6900, Saudi Arabia

^3^ microbiome@UP, Department of Biochemistry, Genetics and Microbiology, Faculty of Natural and Agricultural Sciences, University of Pretoria, Hatfield, Pretoria, 0028

^4^ DSI/NRF SARChI in Marine Microbiomics, Department of Biochemistry, Genetics and Microbiology, Faculty of Natural and Agricultural Sciences, University of Pretoria, Hatfield, Pretoria, 0028

^5^ Scotland’s Rural College, West Mains Road, Edinburgh, EH9 3JG, United Kingdom

^6^ School of Energy, Geoscience, Infrastructure and Society, Heriot-Watt University, Edinburgh, EH14 4AS, United Kingdom

^7^ Mammal Research Institute, University of Pretoria, Private Bag X20, Hatfield, 0028, South Africa ^8^ iThemba LABS, Private Bag 11, WITS, 2050, South Africa

^9^ Department of Biological Sciences, University of Waikato, New Zealand

^10^ Molecular EcoSystems Biology Division, Lawrence Berkeley National Laboratory, 1 Cyclotron Rd, Berkeley, CA, 94720, USA

^11^State Key Laboratory of Tibetan Plateau Earth System and Resources Environment, Institute of Tibetan Plateau Research, Chinese Academy of Sciences, Beijing 100101, China

^12^ Department of Plant Biotechnology and Bioinformatics, Ghent University, 9052 Ghent, Belgium ^13^ Center for Plant Systems Biology, VIB, 9052 Ghent, Belgium

^14^ Bioinformatics Institute Ghent, Ghent University, 9052 Ghent, Belgium

^15^Laboratorio de Biodiversidad y Funcionamiento Ecosistémico, Instituto de Recursos Naturales y Agrobiología de Sevilla (IRNAS), CSIC, Seville, Spain

^16^Unidad Asociada CSIC-UPO (BioFun), Universidad Pablo de Olavide, Seville, Spain

***Corresponding author:**

Thulani P. Makhalanyane Email: [thulani.makhalanyane@up.ac.za](mailto:thulani.makhalanyane@up.ac.za)

This PDF includes:

Supplementary Notes 1-2

Supplementary Figures 1-3

**Supplementary Note 1**

*Soil isotopic ratios and taxonomic diversity*

Carbon stable isotope ratios (δ^13^C range; -23.5 to -31.3‰) were consistent with carbon fixation through the Calvin-Benson-Bassham (CBB) cycle. Nitrogen stable isotope ratios indicated a predominance of *in situ* soil nitrate assimilation processes (δ^15^N range, 2.7 to -11.8‰ ^1^) with evidence of nitrogen fixation at only two sites. Moreover, major cations are important cofactors for many basal cellular processes, such as iron in photosynthesis, and limitations on their abundance may affect soil bacterial communities in this region. For example, available soil K^+^ is directly linked to soil moisture through its role in the movement of water and nutrients in the cell ^2^.

*Cyanobacteria* were rare across all sites, except for samples collected from Pegtop Mountain (PT) and Cliff Nunatak (CN). The high levels of cyanobacterial sequence data from site PT-2 facilitated the reconstruction of an *Aliterella* genome (*Cyanobacteria*; *Chroococcidiopsidales*). The *Aliterella* genome represents the first description of this cyanobacterial genus in continental Antarctica (Pegtop Mountain; PT-2_8), with *A. antarctica* having been recently described in a sample of green turf algae from King George Island, South Shetland Islands ^3^. *A. vladivostokensis* was also isolated from a marine sample in Vladivostok, Russia ^4^. The *Aliterella* PT-2_8 genome encodes a phosphoribulokinase (*prk*) and likely assimilates CO_2_ to produce glucose via the Calvin-Benson-Bassham cycle (*rbcLS*). The release of photosynthate may in turn support heterotrophs at that location. The genome also encodes for siderophore production which is critical to sequester iron - a key co-factor in photosynthesis. Consistently, site PT-2 had the highest soil respiration rate across the region as well as the highest relative abundance of *Cyanobacteria* ^5^.

Both *Verrucomicrobiota* MAGs were G+C rich (mean G+C = 60.6%) and belonged to the order *Chthoniobacterales*. Members of this phylum remain poorly described despite their prevalence in soil, freshwater and deep terrestrial subsurface environments ^6^. These genomes were rich in stress response genes such as multidrug resistance proteins (*mdtC*), ultraviolet repair mechanisms (*uvrABC*) and superoxide dismutases, and encoded the *higBA* toxin-antitoxin module thought to be involved in antibiotic resistance ^7^.

**Supplementary Note 2**

*Uncultivated Viral Genomes in Antarctic soils*

In a survey of the assembled metagenomes for uncultivated viral genomes (UViGs), we found 101 contigs across all assemblies that were of putative viral origin (all > 10 kb) and identified 16 additional prophages. The longest UViG was 37,919 bp and showed sequence similarity to an uncultured *Caudovirales* genome, while the longest prophage genome was 24,114 bp. The soil samples with the most UViGs were Flatiron (*n*=18 UViGs), Mount Seuss 7 (*n*=18) and Mackay Glacier 3 (*n*=16) which is broadly similar to the viral diversity reported from enriched viromes in the same region ^8^. Other samples exhibited only a single UViG. The quality of the viral contigs was assessed using CheckV, which indicated that most contigs were medium- to low-quality. Low-quality contigs are probably genome fragments rather than complete viral genomes, although we identified six high-quality UViGs, including one complete UViG.

We placed our UViGs within the known diversity of bacteriophages by comparing the protein contents of known viral genomes in RefSeq with our UViGs using vContACT2 ^9^. The identified UViGs likely represent novel bacteriophages as all sequences clustered separately from known viral genomes (**Figure 2**). Low levels of protein sequence clustering are common in studies exploring novel phage diversity, and particularly in soils, where up to 97% of viral OTUS (vOTUs) can form separate clusters from known viral sequences ^10^. We clustered our viral contigs in vOTUs based on sequence similarity along the majority of the contigs. A dominant vOTU was detected at nine of the 18 sites (comprising 24 unique UViGs), suggesting the presence of a common bacteriophage across the region. A sequence alignment map showed high sequence similarity levels among these sequences across the nine sampling sites (**Supplementary** **Figure 2**). It is tempting to speculate that aerosol transport of local Antarctic dust particles may contribute to the occurrence of a common vOTU in half of the sites as the most distal sites are ~58 km apart. Microorganisms are relocated by Antarctic wind currents in the Dry Valley region of Eastern Antarctica ^11^ and Antarctic aerosols have longer average residence times in the atmosphere than elsewhere ^12^.

Analyses of the UViG gene content showed the presence of putative auxiliary metabolic genes (AMGs) in some contigs. AMGs are acquired from their cognate bacterial hosts and may have important metabolic functions for phages. For example, cyanophages modulate photosynthesis in the surface ocean via core photosystem II genes acquired from cyanobacteria ^13^. Putative AMGs found in our viral contigs included a glycoside hydrolase 18 (GH18) and a glycosyl transferase 2 (GT2). The GT2 gene was flanked by a transposase supporting the concept that it was acquired by a horizontal gene transfer event. Family GH18 members are chitinases capable of degrading N-acetyl-β-D-glucosamine polysaccharides found in cellular matrices, a process which may assist viral entry into the cell ^14^. We found GH18 genes in four *Bacteroidetes* MAGs (MS4-1_1, MS5-1_6, MS5-1_7 and MS7-5_7) and the *Cyanobacteria* MAG (PT-2_8), all of which showed sequence homology to the viral GH18. Both MS5-1_7 and MS4-1_1 have CRISPR-Cas arrays in their genomes, offering a link between past infections and the transfer of genetic material from the cognate host. It is possible that phages use these genes for the breakdown of lipopolysaccharides of cell membranes which implicate phages in the carbon cycle in this polar desert.

Using VIBRANT ^15^ we discovered four AMGs, namely a chitinase, a ribonuclease H (*rhnA*), a heme synthase (*ahbD*) and a 2-polyprenyl-6-hydroxyphenyl methylase (*ubiG*).

We also characterized prophages in our Antarctic genomes. We found one prophage, in the *Verrucomicrobiota* MS5-1_8 genome, as well as two prophages, in a *Bacteroidota* genome (TG5-1_3). Coincidently, these two taxa were predicted to be the slowest growing. This finding provides direct evidence linking prophages to both *Verrucomicrobiota* and *Bacteroidota* in this region. The prophage found in the *Verrucomicrobiota* genome was 4,483 bp in length and was similar to known *Microviridae* phages (BLASTn: 31% query coverage and 69.74% identity). The prophages identified in the *Bacteroidota* genome were 5,477 bp and 7,214 bp in length, although these had low sequence similarity to known phages. While several studies have investigated the importance of prophages on the evolution of bacterial pathogens ^16^, knowledge regarding their role in soil taxa remains limited and the lack of a comprehensive database of soil-dwelling viruses contributes to this knowledge gap ^17^. Previous studies suggest that lysogenic conversion may confer new traits to bacteria ^18^, which typically have novel metabolic functions through auxiliary metabolic genes (AMGs) ^19^. For example, phages associated with *Verrucomicrobiota* encoded genes have previously been implicated in nitrogen fixation ^20^. In oligotrophic soils, the benefits to the hosts may be vital for ecosystem services, facilitating access to alternative energy sources or stress avoidance mechanisms. Elucidating innate mechanisms may provide insights regarding host-virus interactions and reveal the extent of functional diversity in these soils.

**Supplementary Figure 1. Taxonomic composition and structure of Antarctic soil samples. A)** Relative abundances of prokaryotic and eukaryotic groups across 18 samples from the Mackay Glacier region based on the *rplB* gene. “Other groups” include taxa present as <1% of the total community. **B)** Redundancy analysis overlaid on an nMDS ordination plot with significant soil features overlaid (blue arrows). Soil respiration and major cations (K^+^, Na^+^ and Ca^+^) were significant explanatory variables of community composition. **(C)** Estimates of sequencing depth based on read redundancy as estimated by Nonpareil 3 ^21^.


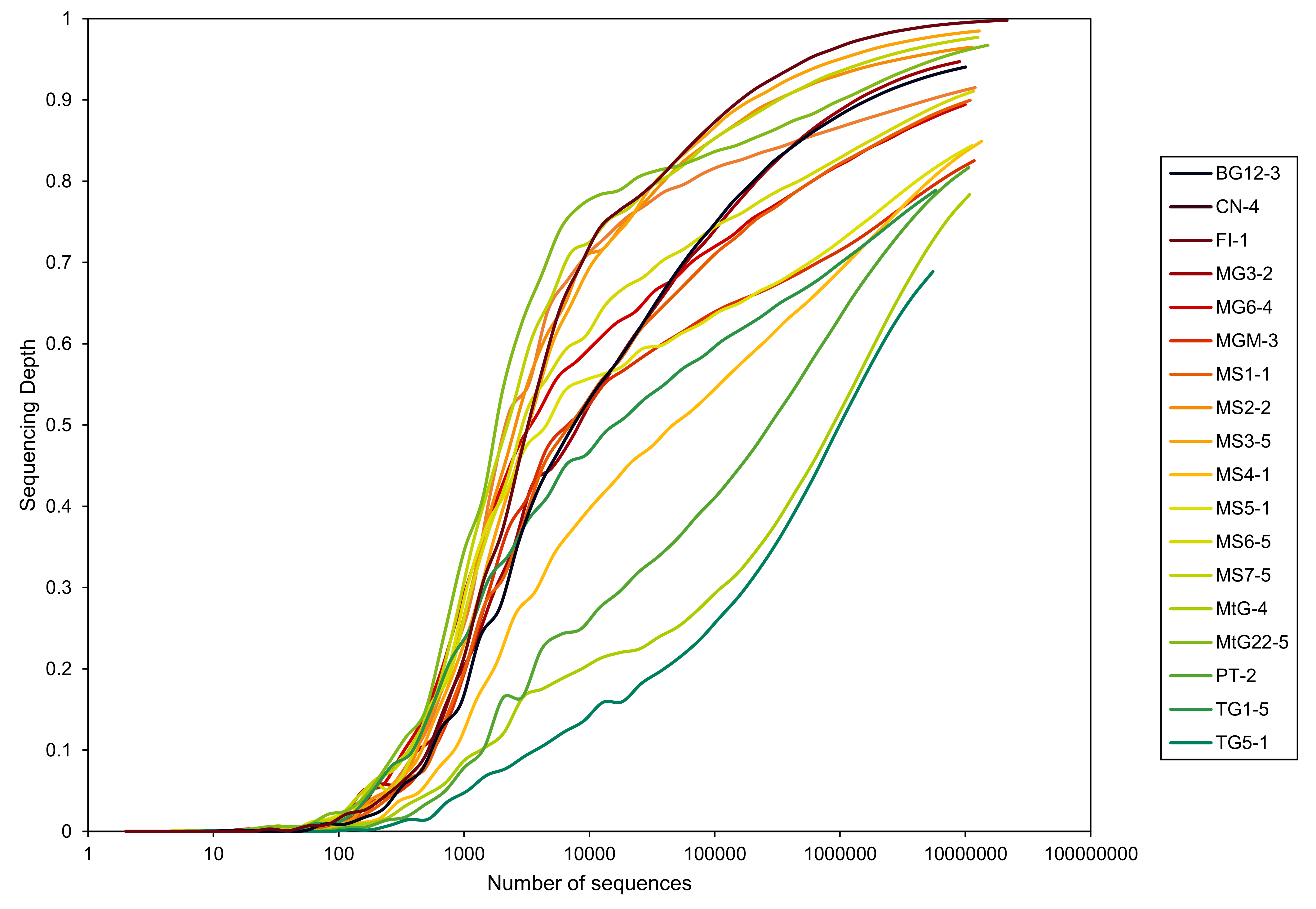

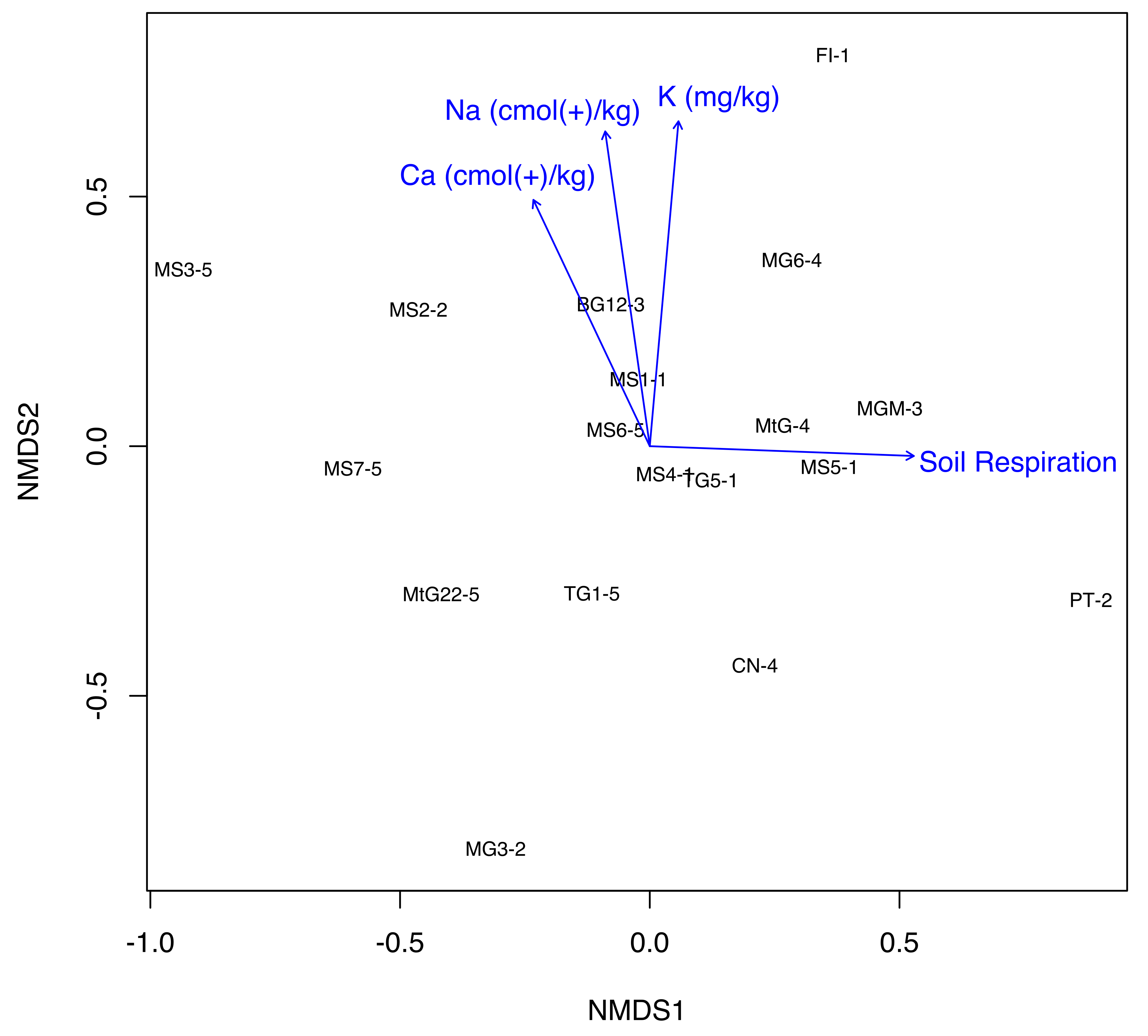

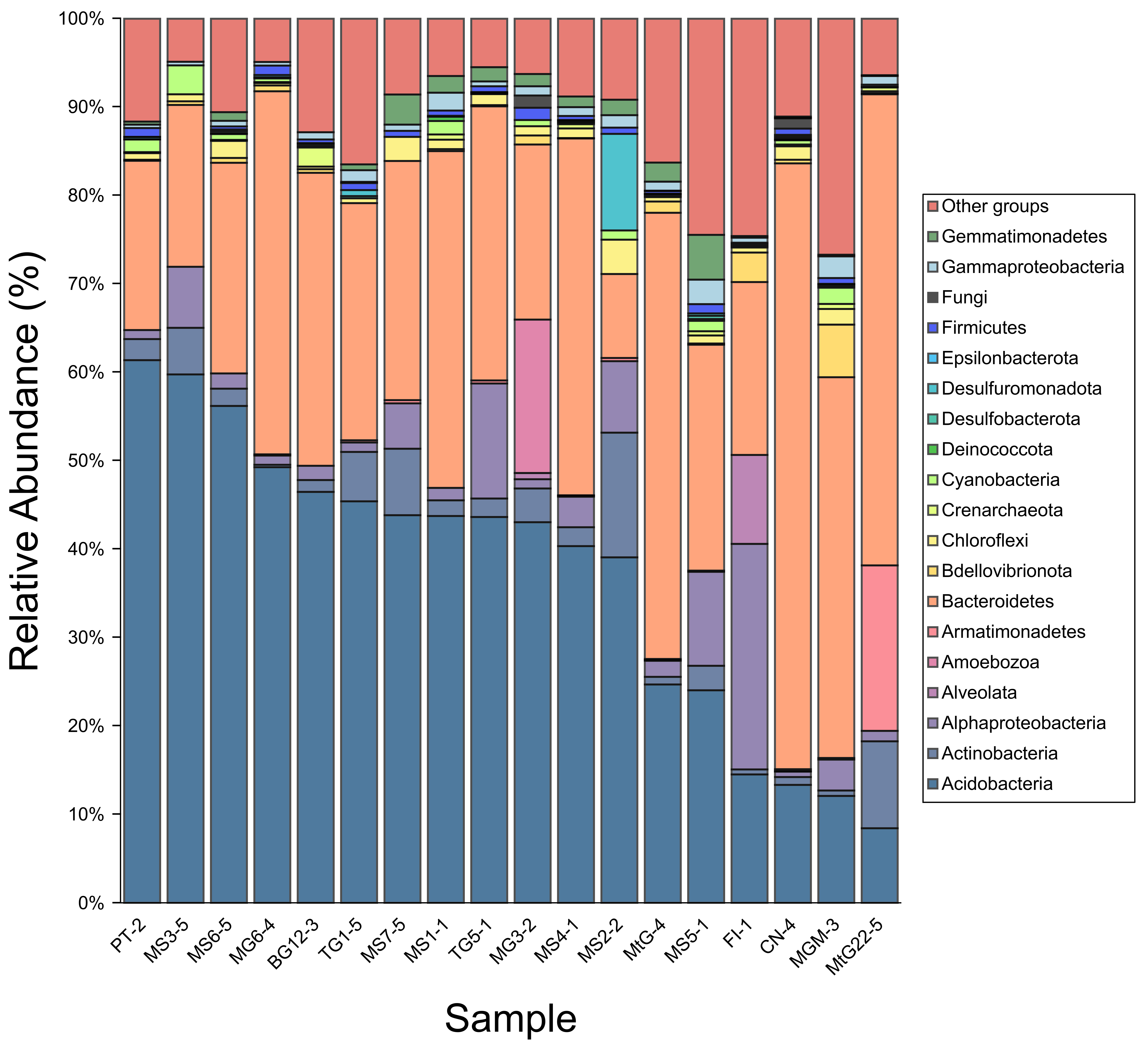


**Supplementary Figure 2. Example of clustering of uncultivated viral genomes (UViGs) into viral OTUs.** The most pervasive vOTU comprises 24 unique viral genomes that spans 11 different sites in this region.

**
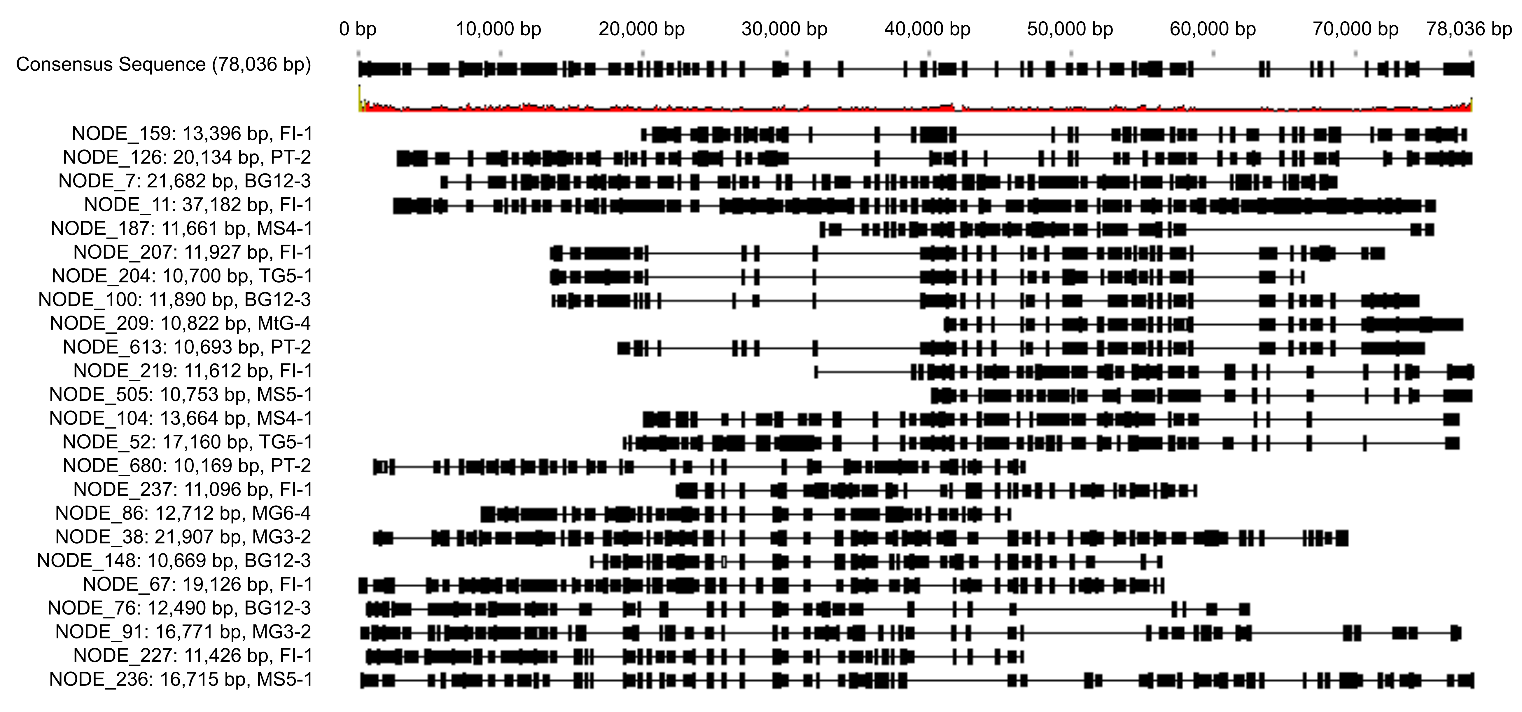
**

**Supplementary Figure 3. Evidence of G+C skew across prophages.**

**
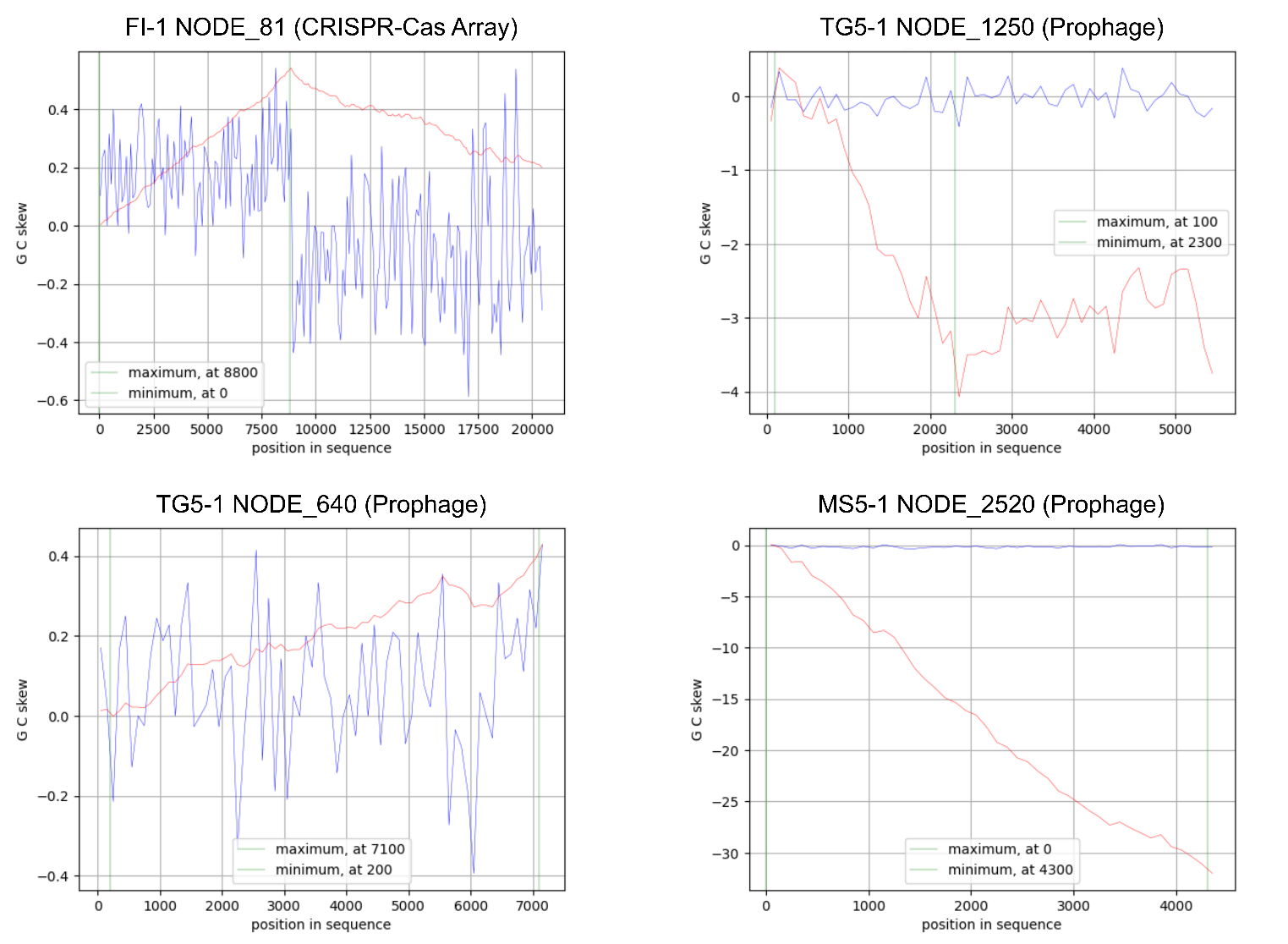
**

**References**

1 Van Goethem, M. W. *et al.* Nutrient parsimony shapes diversity and functionality in hyper-oligotrophic Antarctic soils. *bioRxiv*, 2020.2002. 2015.950717 (2020).

2 Bahadur, I., Maurya, B. R., Kumar, A., Meena, V. S. & Raghuwanshi, R. in *Potassium solubilizing microorganisms for sustainable agriculture* 255-266 (Springer, 2016).

3 Rigonato, J. *et al.* Aliterella atlantica gen. nov., sp. nov., and Aliterella antarctica sp. nov., novel members of coccoid Cyanobacteria. *International journal of systematic and evolutionary microbiology* **66**, 2853-2861 (2016).

4 Abdullin, S. R., Nikulin, A. Y., Bagmet, V. B., Nikulin, V. Y. & Gontcharov, A. A. New cyanobacterium Aliterella vladivostokensis sp. nov.(Aliterellaceae, Chroococcidiopsidales), isolated from temperate monsoon climate zone (Vladivostok, Russia). *Phytotaxa* **527**, 221-233 (2021).

5 Ortiz, M. *et al.* Multiple energy sources and metabolic strategies sustain microbial diversity in Antarctic desert soils. *Proceedings of the National Academy of Sciences* **118**, e2025322118 (2021).

6 Nixon, S. L. *et al.* Genome-resolved metagenomics extends the environmental distribution of the verrucomicrobia phylum to the deep terrestrial subsurface. *Msphere* **4**, e00613-00619 (2019).

7 Armalytė, J., Jurėnas, D., Krasauskas, R., Čepauskas, A. & Sužiedėlienė, E. The higBA toxin-antitoxin module from the opportunistic pathogen Acinetobacter baumannii–regulation, activity, and evolution. *Frontiers in microbiology* **9**, 732 (2018).

8 Adriaenssens, E. M. *et al.* Environmental drivers of viral community composition in Antarctic soils identified by viromics. *Microbiome* **5**, 1-14 (2017).

9 Jang, H. B. *et al.* Taxonomic assignment of uncultivated prokaryotic virus genomes is enabled by gene-sharing networks. *Nature biotechnology* **37**, 632-639 (2019).

10 Li, Z. *et al.* Deep sea sediments associated with cold seeps are a subsurface reservoir of viral diversity. *The ISME Journal*, 1-13 (2021).

11 Bottos, E. M., Woo, A. C., Zawar-Reza, P., Pointing, S. B. & Cary, S. C. Airborne Bacterial Populations Above Desert Soils of the McMurdo Dry Valleys, Antarctica. *Microbial ecology* **67**, 120-128 (2014).

12 Burrows, S. *et al.* Bacteria in the global atmosphere–Part 2: Modeling of emissions and transport between different ecosystems. *Atmospheric Chemistry and Physics* **9**, 9281-9297 (2009).

13 Sullivan, M. B. *et al.* Prevalence and evolution of core photosystem II genes in marine cyanobacterial viruses and their hosts. *PLoS biology* **4**, e234 (2006).

14 Funkhouser, J. D. & Aronson, N. N. Chitinase family GH18: evolutionary insights from the genomic history of a diverse protein family. *BMC evolutionary biology* **7**, 1-16 (2007).

15 Kieft, K., Zhou, Z. & Anantharaman, K. VIBRANT: automated recovery, annotation and curation of microbial viruses, and evaluation of viral community function from genomic sequences. *Microbiome* **8**, 1-23 (2020).

16 Fortier, L.-C. & Sekulovic, O. Importance of prophages to evolution and virulence of bacterial pathogens. *Virulence* **4**, 354-365 (2013).

17 Graham, E. B. *et al.* Global Biogeography of the Soil Virosphere. *bioRxiv*, 2023.2011. 2002.565391 (2023).

18 Bossi, L., Fuentes, J. A., Mora, G. & Figueroa-Bossi, N. Prophage contribution to bacterial population dynamics. *Journal of bacteriology* **185**, 6467-6471 (2003).

19 Jansson, J. K. & Wu, R. Soil viral diversity, ecology and climate change. *Nature Reviews Microbiology* **21**, 296-311 (2023).

20 Tran, P. Q. *et al.* Viral impacts on microbial activity and biogeochemical cycling in a seasonally anoxic freshwater lake. *bioRxiv*, 2023.2004. 2019.537559 (2023).

21 Rodriguez-R, L. M., Gunturu, S., Tiedje, J. M., Cole, J. R. & Konstantinidis, K. T. Nonpareil 3: fast estimation of metagenomic coverage and sequence diversity. *MSystems* **3** (2018).
